# Total Knee Arthroplasty Revision in the Setting of Periprosthetic Joint Infection Resulting in Bone Cement Implantation Syndrome (BCIS), Pulseless Electrical Activity (PEA) Arrest, and Intraoperative Death: A Case Report and Literature Review

**DOI:** 10.7759/cureus.57662

**Published:** 2024-04-05

**Authors:** Cameron Sullivan, Christopher M Russo, Lorelei Wilson, Sean Dennig, Patrick Coleman

**Affiliations:** 1 School of Medicine, Uniformed Services University of the Health Sciences, Bethesda, USA; 2 Anesthesiology, Walter Reed National Military Medical Center, Bethesda, USA; 3 Anesthesiology and Critical Care, Walter Reed National Military Medical Center, Bethesda, USA

**Keywords:** intra-operative, distributive shock, patho-physiology, in-hospital cpr, right ventricular failure, ortho surgery, pea arrest, transesophageal echocardiography (tee), bone cement implantation syndrome

## Abstract

An 87-year-old female with a history of total knee arthroplasty (TKA) presented to the emergency department (ED) for left knee pain in the setting of recent methicillin-sensitive Staphylococcus aureus (MSSA) sepsis of unknown origin. She was subsequently diagnosed with a complicated symptomatic periprosthetic joint infection of her left TKA hardware and was admitted for TKA revision following an orthopedic surgery consultation. Upon arrival at the operating room (OR), standard American Society of Anesthesiology (ASA) monitors were applied. These included non-invasive blood pressure, electrocardiogram (ECG), pulse oximeter, and an esophageal temperature probe. The patient then underwent induction of general endotracheal anesthesia (GETA) without significant hemodynamic compromise. Intraoperatively, the patient tolerated the removal of her infected hardware without major complication but upon placement of the methyl methacrylate (MMA), commonly referred to as *bone cement*, the patient had an acute drop in her end-tidal carbon dioxide (EtCO_2_) and then developed significant bradycardia and hypotension. Despite rapid detection and treatment, the patient continued to collapse hemodynamically and was noted to be pulseless and in pulseless electrical activity (PEA) arrest on ECG. Cardiopulmonary resuscitation (CPR) was immediately started per the Advanced Cardiac Life Support (ACLS) algorithm. Roughly after 45 minutes of continuous CPR and multiple doses of 1 mg epinephrine, it was determined that the patient had suffered a catastrophic and fatal intraoperative event. A team decision was made to stop providing any lifesaving interventions. This patient’s presentation is consistent with bone cement implantation syndrome (BCIS), an uncommon phenomenon that remains poorly understood. Two leading models for BCIS described in the literature are the monomer-mediated and embolus-mediated models. However, further research into BCIS is warranted to better understand its pathophysiology, incidence, as well as potential prophylactic measures, including the use of cementless arthroplasty. This complicated and fatal case serves as a reminder of the morbidity and mortality associated with BCIS and underscores that anesthesiology teams must remain vigilant and prepared during orthopedic joint procedures.

## Introduction

Bone cement implantation syndrome (BCIS) is a poorly understood condition that has been well described to have the potential to result in significant morbidity and mortality. The following is a case of intraoperative death following the use of polymethyl methacrylate (PMMA), also known as bone cement, in a total knee arthroplasty (TKA) revision in the setting of a periprosthetic joint infection. The patient was an 87-year-old female with a medical history significant for hypertension, osteoarthritis, hypothyroidism, and a surgical history of TKA in 2003, with revision in 2010. She presented to the emergency department (ED) for left knee pain and fever. Of note, two weeks before, the patient was diagnosed with methicillin-sensitive Staphylococcus aureus (MSSA) sepsis of unknown origin at a different hospital. The patient's left knee joint was aspirated in the ED and found to have findings consistent with periprosthetic joint infection and was admitted to the inpatient service for further evaluation. Preoperative diagnostic imaging studies of her left knee confirmed the presence of infected hardware, an orthopedic surgeon was consulted and recommended a TKA revision. The patient underwent a left adductor canal (AC) peripheral nerve block without complication before the start of the operation and was subsequently prepped for surgery.

## Case presentation

**Figure 1:**
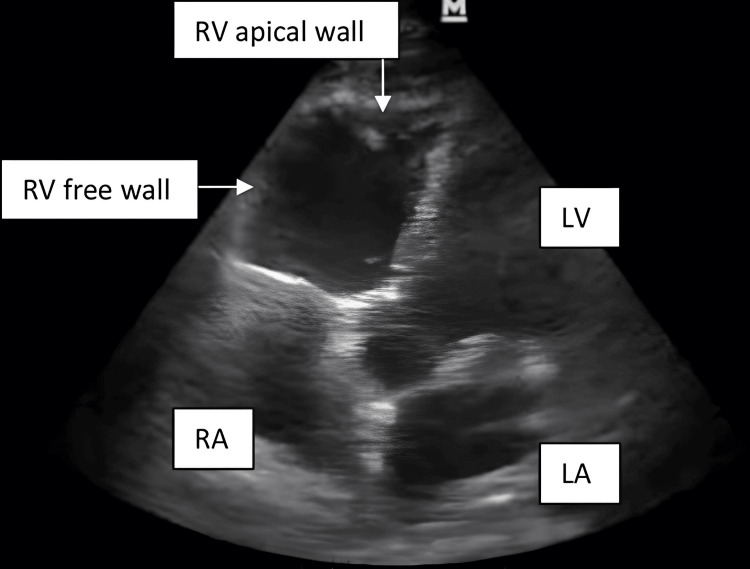
Hypokinetic and severely dilated RV in the setting of acute RV failure secondary to BCIS visualized intraoperatively on TTE via apical 5 chamber view. RV, right ventricle; BCIS, bone cement implantation syndrome; TTE, transthoracic echocardiography; RA, right atrium; LV, left ventricular; LA, left atrium

An 87-year-old female with recent MSSA sepsis of unknown origin presented to the ED with left knee pain and underwent diagnostic left knee joint aspiration with cytology that was suggestive of infected hardware. The patient was admitted preoperatively in stable condition and underwent further imaging studies confirming the diagnosis resulting in an orthopedic surgery consult for left TKA revision. On the morning of surgery, the patient was appropriately NPO and had no personal or family history of complications with anesthetics in the past. The patient underwent an uncomplicated preoperative nerve block of her left AC peripheral nerve with 20 mL of 0.5% ropivacaine. Upon arriving at the operating room (OR), standard American Society of Anesthesiology (ASA) monitors were applied, while the patient was adequately preoxygenated with 100% FiO_2_ via an anesthetic face mask. Induction of anesthesia was performed with lidocaine, propofol, fentanyl, and rocuronium without any significant hemodynamic complications, and direct laryngoscopy was achieved via Macintosh size 3 laryngoscope blade and was successfully intubated in a single attempt with a 7.0 endotracheal tube (ETT). Maintenance of anesthesia was achieved with a 1.0 minimum alveolar concentration (MAC) of sevoflurane. Intraoperatively, the patient tolerated the removal of her infected hardware without major complications. Upon placement of the methyl methacrylate (MMA), commonly referred to as *bone cement*, the patient had an acute drop in her end-tidal carbon dioxide (EtCO_2_) on continuous waveform capnography from 35-45 to 15-20 mmHg. After ensuring the ETT had not inadvertently moved via lung auscultation and the ventilator circuit was not leaking, it was determined by the anesthesiology team that this acute reduction in EtCO_2_ was likely representing an acute reduction in cardiac output (CO). About a minute later, the patient developed significant bradycardia with a heart rate of 33 (previously 60-85) and hypotension of 80/40. The surgical team was immediately notified of the acute hemodynamic change, and surgery was halted while the anesthesiology team rapidly worked to increase the patient’s blood pressure and CO with 1 mg atropine and 10 mg ephedrine boluses. Despite the rapid detection and treatment, the patient continued to rapidly collapse hemodynamically and was noted to be pulseless and in pulseless electrical activity (PEA) arrest on ECG. The medical director and on-call anesthesiology team were urgently paged to the intra-op, the surgical drapes were dropped, and CPR was initiated per the 2023 Advanced Cardiac Life Support (ACLS) protocol by the anesthesiology team. Defibrillator pads were attached to the patient while the team continued CPR, and 1 mg of epinephrine was administered. The patient would transiently regain carotid pulses but would become pulseless shortly after that. The Surgical Intensive Care Unit (SICU) and cardiothoracic anesthesiology services arrived while the primary anesthesiology team rapidly gained critical vascular access via arterial and central venous lines. Transesophageal echocardiography (TEE) was performed during CPR and showed severe right ventricular (RV) hypokinesis and dilation with minimal tricuspid valve motion and no motion of the RV-free wall (Figure 1). Furthermore, left ventricular (LV) function was noted to appear severely depressed and showed negligible contractility with no evidence of pulmonary embolism, pericardial effusion, or valvular lesion. After approximately 45 minutes of continuous CPR including multiple doses of 1 mg epinephrine in conjunction with the intra-code TEE findings, it was determined that the patient had suffered a catastrophic and fatal intraoperative event, and a team decision was made to stop providing any lifesaving interventions.

## Discussion

PMMA, sometimes referred to as MMA or *bone cement*, was first used in orthopedic surgery in 1965 and later received approval from the U.S. Food and Drug Administration (FDA) in 1970 [1]. In 1970, Powell et al. were among the first to describe bone cement’s association with acute cardiac arrest when they reported two instances of intraoperative cardiac arrest following the replacement of a femoral head with a Thompson prosthesis [2]. Despite early catastrophic descriptions of BCIS, a comprehensive etiology and pathophysiology remains unknown. There are, however, many proposed mechanisms reported in the literature. Of the many hypothesized mechanisms of BCIS, the two leading models are the monomer-mediated model and the embolus-mediated model. This research shows that a combination of both models may contribute to acute hemodynamic cardiovascular changes in a synergistic fashion. The first and classically accepted model is the monomer-mediated model. This mechanism proposes that when activated for use in orthopedic surgery, MMA results in a very strong exothermic reaction that results in the rapid expansion of the bone cement within the desired bone cavity in which it is placed. It has been postulated that MMA has very strong direct cardiopulmonary depressant effects and is rapidly absorbed into the bloodstream via the bone marrow, resulting in a rapid and noxious bolus to the heart and lungs once placed in the patient [1]. In vitro studies demonstrated direct vasodilatory effects of MMA; however, in vivo animal studies failed to demonstrate sufficient plasma concentration of MMA following cemented hip arthroplasty to achieve the significant cardiopulmonary effects commonly seen in BCIS [1,3]. This discrepancy led to the more recently developed and proposed embolus-mediated model. The proposed mechanism of the embolus-mediated model suggests that the acute and seemingly unpredictable cardiopulmonary effects are more likely to be caused by high mechanical intramedullary pressures (>300 mmHg) due to the rapid and expansive exothermic reaction due to MMA, as mentioned earlier, in the setting of existing components with embolic potential (fat particles, bone particles, microthrombi, MMA, platelets, fibrin, and other tissue breakdown products). The aforementioned particles are rapidly absorbed into the bone marrow due to a combination of high intramedullary chemical pressure (resulting from the exothermic reaction) and mechanical pressures applied during the cementing process of the new prosthesis into its desired location. This well-known phenomenon results in the observable *showering *of emboli to the right heart and subsequently to the lungs, which is characteristically seen in intraoperative TEE in cases of BCIS [1]. These showers of pulmonary emboli (and bone particles) are thought to cause hypoxia and RV dysfunction, ultimately leading to hypotension, characteristic of BCIS. The presence of pulmonary emboli is also thought to play a role in mediator release, contributing to the increased pulmonary vascular resistance, further worsening RV function, resulting in an acute decline in RV ejection fraction (RVEF) [4]. Intraoperatively, this embolism-induced cascade initially presents as a sudden decrease in EtCO_2_ [5]. First proposed in 2009 by British Anesthesiologist Donaldson et al., the criteria for the four different grades (Grades 0-3) were developed based on hypoxia, hypotension, loss of consciousness, and the requirement of cardiopulmonary resuscitation (CPR). The criteria and their respective incidence (as of 2014) can be seen in the charts below [4,6]. Using the Donaldson criteria, Olsen et al. estimated the incidence of BCIS in cemented hemiarthroplasty for femoral neck fractures to be 21%, 5.1%, and 1.7% for BCIS grades 1, 2, and 3, respectively [7]. Still, the overall incidence of BCIS is poorly understood due to the significant range in clinical presentation as well as the type of arthroplasty [1]. Specific risk factors can greatly increase the incidence of BCIS such as preexisting pulmonary hypertension, New York Heart Association class 3 or 4, Canadian Heart Association class 3 or 4, pathologic fractures, inter-trochanteric fractures, long-stem arthroplasty, and a history of malignancy. Schwarzkopf et al. demonstrated this increased incidence in their retrospective study where some observable degree of BCIS occurred in 75% of cancer patients undergoing hip arthroplasty [1,8]. To mitigate the risk of BCIS in patients with known risk factors, several prophylactic measures have been described including installation of prostheses without the use of bone cement [9]. Rates of cementless arthroplasty vary by country. Recently in the United States, 93% of primary total hip arthroplasties are cementless, whereas uncemented rates vary among other nations [10]. In the past decade from 2010 to 2017, rates of uncemented fixation increased in Sweden, Denmark, and Norway but decreased in England-Whales, Australia, New Zealand, and Finland [11]. Further research into BCIS is warranted to better understand its pathophysiology, its incidence, as well as potential prophylactic measures to include cementless arthroplasty.

## Conclusions

The aforementioned case describes a rare but fatal example of a complication of BCIS resulting in PEA arrest and intraoperative death in a patient undergoing cemented arthroplasty revision. While the etiology of BCIS is not fully understood, the monomer-mediated model and embolus-mediated model represent the two leading hypotheses for the range of acute clinical findings and may both be synergistically implicated in BCIS. Intraoperatively, a sudden decrease in EtCO_2_ is an early indicator of the decrease in cardiac output, which ultimately may lead to hypoxia, hypotension, and cardiac arrest. However, TEE is essential for early detection and treatment. This complicated and fatal case serves as a reminder of the morbidity and mortality associated with BCIS and underscores that both the orthopedic surgical and anesthesiology teams must remain vigilant and prepared during orthopedic joint procedures involving the use of PMMA, MMA, or bone cement.
